# Exophytic Gastric Mass Posing a Diagnostic Dilemma: A Case of Mesenteric Fibromatosis

**DOI:** 10.7759/cureus.71915

**Published:** 2024-10-20

**Authors:** Alekhya Madisetty, Mohana Priya, Kishor R J, Mahin Nallasivan, Arumugam Vasugi

**Affiliations:** 1 Department of General Surgery, Sri Ramachandra Institute of Higher Education and Research, Chennai, IND; 2 Department of Pathology, Sri Ramachandra Institute of Higher Education and Research, Chennai, IND

**Keywords:** differential diagnosis, exophytic gastric mass, gastrointestinal stromal tumours, mesenteric fibromatosis, multiple episodes of vomitings

## Abstract

Mesenteric fibromatosis (MF) and gastrointestinal stromal tumor (GIST) are distinct types of lesions that are often mistaken for one another. Fibromatosis of the stomach is a rare condition that can be misdiagnosed as GIST, with MF accounting for only 0.3% of all tumors. Due to its rarity, accurately identifying and appropriately treating this condition poses a significant challenge. Here, we report a case of a 45-year-old female who presented with multiple episodes of vomiting after food intake. Initial radiological investigations suggested a diagnosis of GIST. However, histopathological examination of the surgical excision specimen confirmed fibromatosis with a keloid pattern. Beta-catenin is a key marker for MF; although immunohistochemistry markers were negative for beta-catenin in this case, it is important to note that beta-catenin positivity is observed in only 70-80% of cases. Differentiating these two distinct tumors can be challenging due to potential overlaps in clinical presentation, macroscopic appearance, and even histological features.

## Introduction

Mesenteric fibromatosis (MF) is a rare, non-metastasizing type of fibromatosis, also known as an intra-abdominal desmoid tumor, that arises from the mesentery. This locally aggressive tumor consists of a benign growth of fibroblastic and myofibroblastic cells, typically exhibiting a uniform growth pattern of spindle-shaped cells without atypia. The condition is associated with collagen deposition, often of the keloid type [1].

Clinically, MF often presents as an abdominal mass, accompanied by symptoms such as pain and intestinal obstruction, which may vary depending on the size and location of the tumor. Diagnosis is typically made using CT or MRI imaging, with surgery being the preferred treatment option. However, recurrence is common, necessitating ongoing monitoring. Despite its local aggressiveness, the overall prognosis is favorable due to its nonmetastatic nature.

## Case presentation

**Figure 1:**
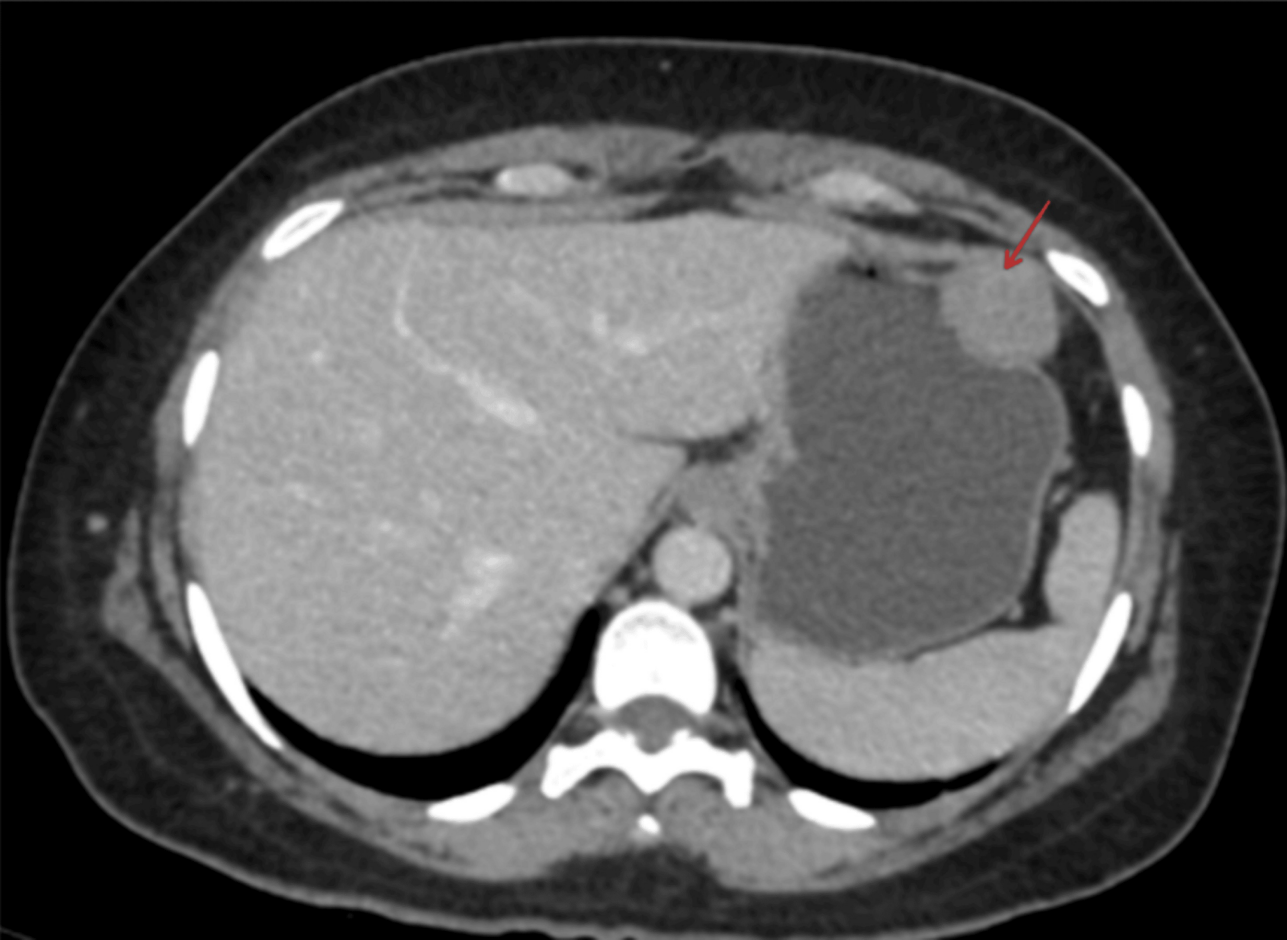
CECT findings of the abdomen In the CECT scan, the red arrow indicates the extraluminal mass originating from the greater curvature of the stomach. CECT, contrast-enhanced CT

A 45-year-old female was admitted to the hospital with a primary complaint of multiple vomiting episodes. The vomit primarily consisted of food particles and was neither blood-stained nor bile-stained, accompanied by dyspepsia. Routine investigations were performed, and an upper gastrointestinal endoscopy revealed antral gastritis with a positive result for the rapid urease test. Subsequently, a contrast-enhanced CT scan of the abdomen was performed, revealing a well-defined, homogeneous extraluminal solid mass measuring 2.9 cm × 3.4 cm × 3.9 cm. This mass originated from the greater curvature of the stomach and showed no significant contrast enhancement, calcification, hemorrhage, or necrosis. Additionally, there was no evidence of infiltration into adjacent structures (Figure 1).

**Figure 2:**
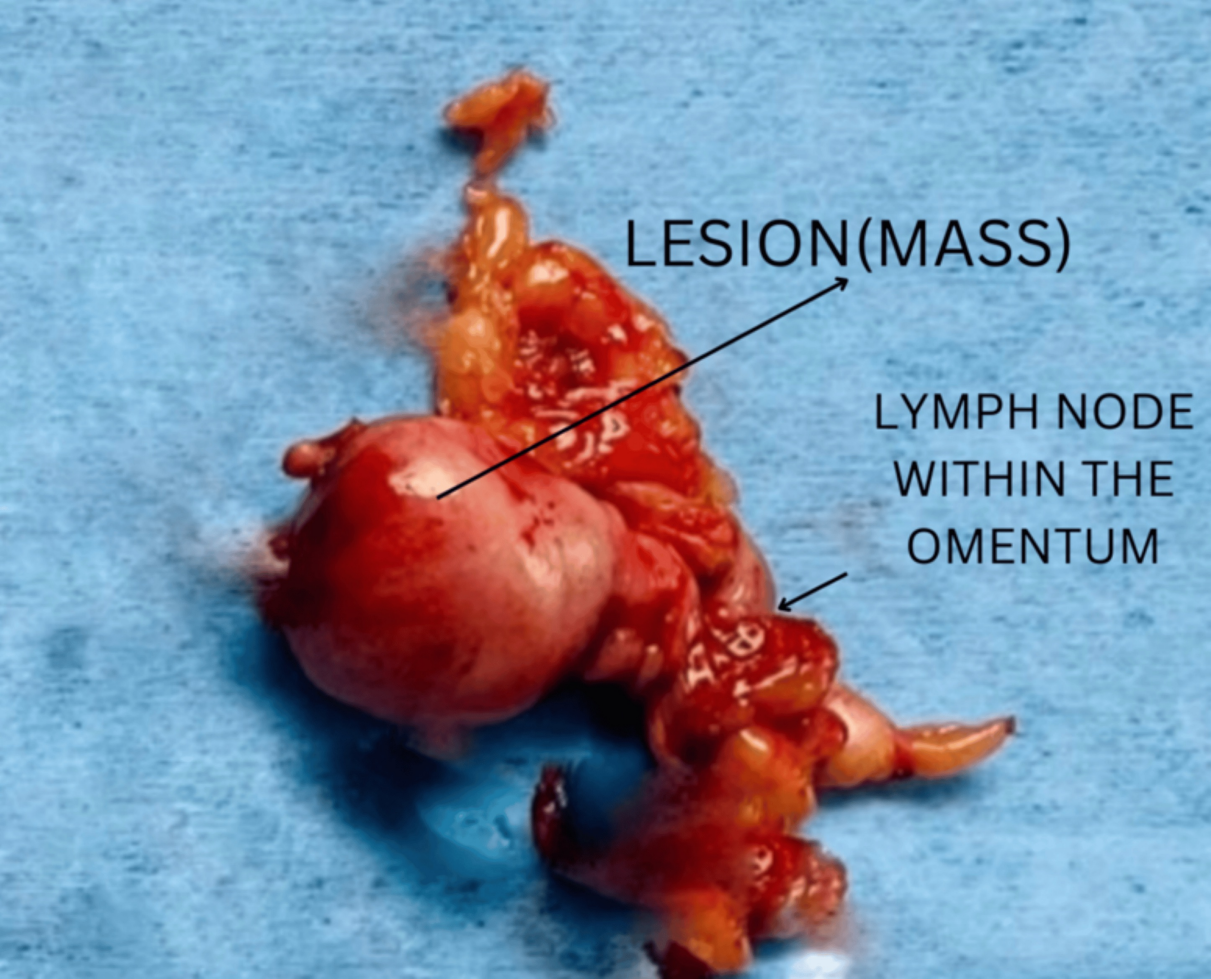
Surgical specimen following wide local excision

The mass was initially suspected to be a gastrointestinal stromal tumor (GIST) due to its extraluminal location arising from the greater curvature of the stomach. The patient subsequently underwent laparoscopic excision. During the procedure, an extraluminal exophytic gastric mass measuring 4 cm × 3 cm × 3 cm was identified. The mass was pedunculated and adhered to the surrounding omentum (Figure 2).

A small subcentimetric lymph node was observed near the lesion. Both the exophytic gastric mass and the lymph node were excised and sent for histopathological examination. Grossly, the mass was a well-circumscribed lesion located in the submucosa, exhibiting a smooth external surface, with a cut surface appearing gray-white and firm in consistency.

**Figure 3:**
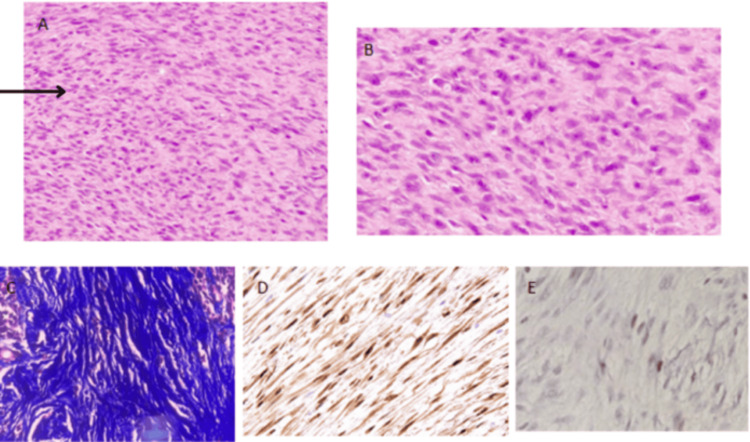
Histopathological images of the specimen (A) H&E stain showing a spindle cell neoplasm intermixed with lymphoid aggregates and occasional plasma cells (H&E, 200x). Spindle cells are indicated by the arrow. (B) H&E stain revealing a spindle cell neoplasm with similar features as in (A) (H&E, 400x). (C) Masson’s trichrome stain highlighting collagen bundles in blue (MT, 400x). (D) Immunohistochemical staining for beta-catenin demonstrating negativity in spindle cells (IHC, 200x). (E) IHC for Ki67, indicating a labeling index of 2% (IHC, 400x). IHC, immunohistochemistry

Microscopic examination revealed a well-circumscribed neoplasm composed of spindle cells intermixed with nests of lymphoplasmacytic infiltrate, hyalinized blood vessels, and ropy collagen (Figure 3A, 3B). The spindle cells were arranged in bundles and storiform patterns. Individual cells exhibited indistinct cell borders and oval to spindle-shaped vesicular nuclei with powdery chromatin. There was no evidence of overt cytological atypia, necrosis, or increased mitotic activity. The lymph node specimen showed only fibroadipose tissue, with no lymphoid tissue identified. Special staining with Masson's trichrome highlighted the collagen bundles within the neoplasm (Figure 3C).

The histological differentials for this case include fibromatosis, GIST, inflammatory myofibroblastic tumor, and spindle cell tumors of mesenchymal lineage. To narrow down the diagnosis, immunohistochemistry (IHC) was performed. The neoplastic cells tested positive for vimentin, with a low Ki-67 labeling index of only 2%, indicating low proliferative activity. Additionally, the cells were negative for smooth muscle actin (SMA), S-100, CD-117, DOG-1, ALK, STAT-6, and IgG4, which helped rule out other differential diagnoses.

The IHC results for our case are presented in Table 1.

**Table 1:** Immunohistochemical expression patterns of neoplastic cells IHC, immunohistochemistry

Positive IHC markers	Negative IHC markers
Vimentin Ki-67 LI-2% (Figure 3E)	SMA, S100, IgG4, STAT-6, CD117, DOG-1, beta-catenin (Figure 3D), and ALK

Histopathologic analysis confirmed the diagnosis of MF. The patient was followed up for six months after surgery and did not report any recurrence of symptoms.

## Discussion

MF, also known as mesenteric desmoids, represents locally aggressive lesions that can infiltrate surrounding tissues and have a propensity for recurrence without metastasizing. This condition predominantly affects females, accounting for approximately 80% of cases, typically occurring between the ages of 14 and 75, with a mean age of 41 years. There is no racial preference associated with MF. The higher prevalence among females is largely attributed to the influence of estrogen, including exogenous sources, which contributes to the development of these tumors [2].

The presentation of MF varies based on its location, often appearing as a soft tissue mass on CT. It may resemble GISTs, which are mesenchymal neoplasms of the digestive tract. We present a case of MF that posed a diagnostic challenge. On CT, MF typically appears as a well-defined homogeneous mass, while GISTs are generally visualized as well-defined heterogeneous masses characterized by a peripheral solid rim that enhances with contrast and a central region exhibiting fluid attenuation due to necrosis, hemorrhage, or myxoid degeneration [3]. In our case, the mass presented as a homogeneous mass with no signs of calcification or necrosis, suggesting MF.

Both GISTs and MF show positive staining for vimentin when analyzed using immunohistochemical markers. The CD117 (c-KIT proto-oncogene product) protein is the most specific and significant immunohistochemical marker for GISTs. A study found that GISTs consistently exhibited diffuse positivity for CD117 (11 of 11) and CD34 (11 of 11), with variable actin expression (focal in five of 11), and were negative for desmin, keratin, and S-100 protein. In contrast, MF displayed CD117 positivity in six of 10 cases (three focal and three diffuse), generally at a weaker intensity compared to GISTs, as well as focal actin expression in five of nine cases and focal desmin in three of eight cases, indicating myofibroblastic differentiation. Notably, MF lacked expression of CD34, S-100, and keratin. While GISTs do not express beta-catenin, this marker is detected in MF, typically in 70-80% of cases [4]. Our case falls within the remaining 20%, characterized by negative beta-catenin staining despite being diagnosed as MF.

The Ki-67 protein is associated with cellular proliferation, and a Ki-67 labeling index of less than 2% indicates low proliferative activity, characteristic of a grade 1 neoplasm, suggesting a slower tumor growth rate. Vimentin is a marker for mesenchymal cells, which is positive in mesenchymal tumors. SMA is expressed in smooth muscle cells and myofibroblasts, as well as tumors with smooth muscle differentiation, such as leiomyoma and leiomyosarcoma. S-100 is a marker for neural crest-derived cells, while IgG4 is associated with IgG4-related diseases that involve fibroinflammatory lesions. STAT-6 is utilized in the diagnosis of solitary fibrous tumors, and CD-117 and DOG-1 are markers specific to GISTs. Negative results for SMA, S-100, CD-117, DOG-1, ALK, STAT-6, and IgG4 further reinforced the diagnosis of MF, ruling out other potential differential diagnoses.

A study has reported that intra-abdominal fibromatosis frequently arises following tumor resection. Years after tumor removal, a mass is often detected, initially believed to be a recurrence of the original tumor; however, it is later identified as MF rather than a true recurrence [5]. Similarly, another study reported two cases initially diagnosed as lymph node metastasis, later revealed to be MF [6]. These examples highlight that MF is often misdiagnosed as another tumor or lesion. It is crucial to recognize that MF is a significant diagnosis and should always be considered during evaluation, as likely as other potential possibilities.

## Conclusions

This case is unique in that MF presents as a growth originating from the greater curvature of the stomach, with vomiting as the primary symptom. Notably, unlike most cases of MF, this one did not stain positive for beta-catenin, adding another layer of rarity to the case. When evaluating an intra-abdominal mass, the differential diagnosis typically includes adenocarcinomas, lymphomas, GISTs, metastases, and other malignancies, while MF is rarely considered initially. This case underscores the importance of recognizing that MF can manifest in various forms and should always be kept in mind as a possible diagnosis.

The diagnosis of MF should be considered due to its potential for local aggressiveness and high recurrence rates. Accurate diagnosis is crucial, as misdiagnosis can result in inappropriate treatment plans and potentially worsen patient outcomes. Early identification and proper management are essential for improving long-term prognosis.
